# The Amino-Terminal Part of the Needle-Tip Translocator LcrV of *Yersinia pseudotuberculosis* Is Required for Early Targeting of YopH and *In vivo* Virulence

**DOI:** 10.3389/fcimb.2016.00175

**Published:** 2016-12-05

**Authors:** Sofie Ekestubbe, Jeanette E. Bröms, Tomas Edgren, Maria Fällman, Matthew S. Francis, Åke Forsberg

**Affiliations:** ^1^Laboratory for Molecular Infection Medicine Sweden, Department of Molecular Biology, Umeå UniversityUmeå, Sweden; ^2^Department of Molecular Biology, Umeå Centre for Microbial Research, Umeå UniversityUmeå, Sweden; ^3^Department of Clinical Microbiology, Umeå UniversityUmeå, Sweden

**Keywords:** LcrV, type III secretion system, YopH, translocation, pore formation, *Yersinia pseudotuberculosis*, virulence

## Abstract

Type III secretion systems (T3SS) are dedicated to targeting anti-host effector proteins into the cytosol of the host cell to promote bacterial infection. Delivery of the effectors requires three specific translocator proteins, of which the hydrophilic translocator, LcrV, is located at the tip of the T3SS needle and is believed to facilitate insertion of the two hydrophobic translocators into the host cell membrane. Here we used *Yersinia* as a model to study the role of LcrV in T3SS mediated intracellular effector targeting. Intriguingly, we identified N-terminal *lcrV* mutants that, similar to the wild-type protein, efficiently promoted expression, secretion and intracellular levels of Yop effectors, yet they were impaired in their ability to inhibit phagocytosis by J774 cells. In line with this, the YopH mediated dephosphorylation of Focal Adhesion Kinase early after infection was compromised when compared to the wild type strain. This suggests that the mutants are unable to promote efficient delivery of effectors to their molecular targets inside the host cell upon host cell contact. The significance of this was borne out by the fact that the mutants were highly attenuated for virulence in the systemic mouse infection model. Our study provides both novel and significant findings that establish a role for LcrV in early targeting of effectors in the host cell.

## Introduction

Type Three Secretion systems (T3SS) were discovered more than 25 years ago when it was established that *Yersinia* spp. targeted virulence proteins (effectors) into eukaryotic cells through a delivery mechanism that required close bacteria-host cell contact (Rosqvist et al., 1991, 1994; Sory and Cornelis, 1994). The T3SS require the coordination of more than 20 genes to promote expression and secretion of the virulence proteins from the bacterium (Galán and Wolf-Watz, 2006).

Over the years, T3SS have been described in a wide variety of gram negative pathogens. While some of them, such as *Salmonella* spp. and *Shigella* spp., use their T3SS to promote uptake by host cells (Finlay et al., 1988; Sasakawa et al., 1988, 1989; Elsinghorst et al., 1989), in other pathogens such as *Yersinia* spp. and *Pseudomonas* spp., the T3SS act to block uptake (Rosqvist et al., 1988; Frithz-Lindsten et al., 1997). These opposing outcomes are ascribed to the specific enzymatic activities and corresponding molecular targets of the translocated effectors, rather than the actual T3SS structure and function that is overall well conserved (Rosqvist et al., 1995; Frithz-Lindsten et al., 1997, 1998; Akopyan et al., 2011).

The injection model is widely used to explain the mode of function of the T3SS. It dictates that protein translocation occurs in one step from the bacterial cytosol to the target-cell cytoplasm through a conduit created by the basal body and a needle-like hollow tube that is extended by a tip complex that forms a pore in the host cell membrane (Galán and Wolf-Watz, 2006). While there is evidence from recent elegant studies that the T3SS substrates are indeed secreted through the narrow hollow needle complex (Dohlich et al., 2014; Radics et al., 2014), there is to date no direct experimental evidence that effectors secreted via the needle are subsequently targeted directly into the host cell. On the contrary, a recent study showed that effectors exogenously added to the bacterial surface of both *Yersinia* and *Salmonella* could be translocated in a T3SS-dependent manner, suggesting an alternative mechanism to the one-step injection model (Akopyan et al., 2011).

The secreted substrates can be divided into two functional classes; effectors and translocators. The effectors are delivered into the target cell where they elicit a specific biological response. *Yersinia* is an extracellular pathogen that replicates in the lymphatic tissues of the host (Hanski et al., 1989; Simonet et al., 1990). As such, *Yersinia* needs to be able to block phagocytosis by the host immune cells such as macrophages, and the effector YopH is essential for this event (Rosqvist et al., 1988; Fahlgren et al., 2009). Phagocytosis is preceded by formation of focal adhesion sites, which occurs at the cytoplasmic side of the host cell membrane. Since phagocytosis is a rapid process with an onset immediately after the establishment of bacteria-cell contact, translocation and targeting of the effectors must essentially occur instantly. Consistent with this, studies have shown that YopH is translocated and targeted to the intracellular focal adhesion sites within minutes after target cell contact (Andersson et al., 1996, 1999; Persson et al., 1999), and that the T3SS substrate YopK is involved in YopH targeting to the focal adhesion sites (Thorslund et al., 2011; Dewoody et al., 2013). The translocators facilitate the delivery of effectors across the plasma membrane (Rosqvist et al., 1994; Sory and Cornelis, 1994; Håkansson et al., 1996; Pettersson et al., 1999). The T3SS encode three functionally conserved translocator proteins, two that are generally hydrophobic and one that is hydrophilic (Matteï et al., 2011). A key signature of the hydrophobic translocators is their putative membrane spanning domain(s), suggesting that they may target host membranes. Indeed, in *Yersinia* spp. the hydrophobic translocators, YopB, and YopD, have been shown to insert into erythrocyte membranes and induce pore formation (Rosqvist et al., 1994; Sory and Cornelis, 1994). LcrV, the hydrophilic translocator, localizes at the tip of the needle complex (Mueller et al., 2005). LcrV facilitates insertion of YopB and YopD in host cell membranes (Broz et al., 2007) and has been proposed to promote translocation by serving as a pore-forming platform. In addition, LcrV also has a role in regulation of *yop* expression since deletion of *lcrV* results in down-regulated Yop production (Price et al., 1991; Pettersson et al., 1999).

We have previously shown that LcrV secretion levels correlates to *in vivo* virulence of *Yersinia* (Bröms et al., 2007). As for other T3SS substrates, the very N-terminal region of LcrV is somehow recognized by the secretion system and, thus, mutants impaired for secretion have been mapped to the N-terminal region (Bröms et al., 2007). Interestingly, introduction of frame-shift mutations that completely changed the coding sequence of the extreme N-terminal of LcrV, affected neither LcrV secretion nor overall expression/secretion of other Yop proteins. These LcrV mutants also induced YopE mediated cytotoxicity in infected HeLa cells to the same extent as the isogenic wild type strain (Bröms et al., 2007). In this study, we performed a more in depth analysis of the N-terminal mutant strains and could hereby show a novel role for the N-terminal region of LcrV in the early targeting of Yop effectors, and the corresponding ability of *Yersinia* to block phagocytosis by macrophages and promote virulence in mice.

## Results

To study the role of LcrV in translocation we decided to utilize two previously characterized in-*cis* N-terminal mutants of LcrV (Bröms et al., 2007). These mutants contain +1 or −1 frame-shift mutations, specifically altering the sequence of the first 15 amino acids, but leaving the remaining protein sequence intact (Figure S1) and will hereafter be referred to as LcrV+1 and LcrV−1 respectively. Importantly, both mutants exhibit intact Yop-regulation, which is a prerequisite to meaningfully study any other role of LcrV.

### The LcrV N-terminus is important for pore formation in erythrocytes

**Figure 1:**
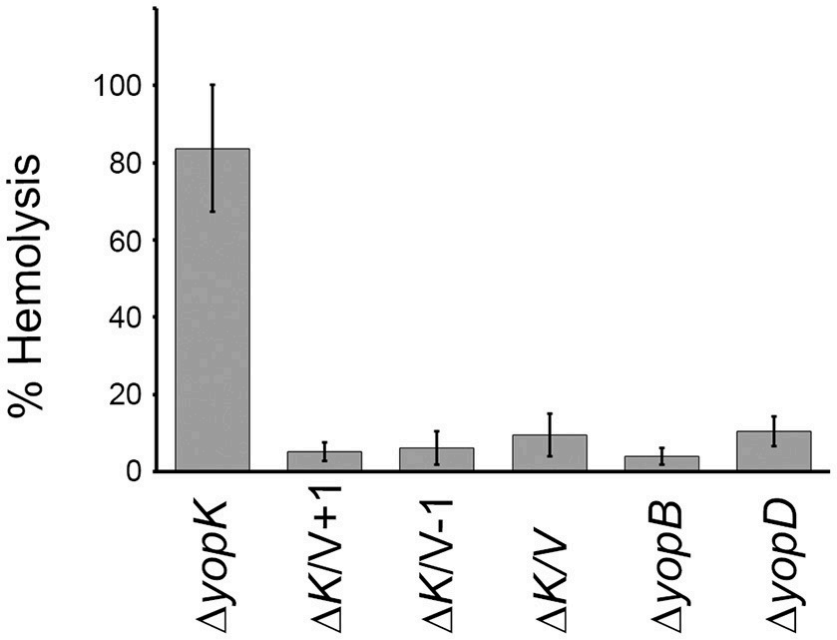
**The LcrV frameshift variants are non-hemolytic**. Sheep erythrocytes were infected with *Y. pseudotuberculosis* strains for 2 h in 96-well plates. The cells were centrifuged and the supernatants collected. The lytic activity was measured as absorbance at 570 nm and is presented as percent of complete lysis (erythrocytes lysed with 1% Triton X-100). The results shown are the mean values ± SD of four independent experiments.

**Figure 2:**
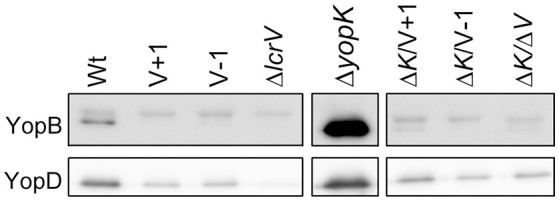
**The extreme N-terminal of LcrV is required for membrane insertion of YopB and YopD**. Sheep erythrocytes were infected with *Y. pseudotuberculosis* for 2 h. The cells were lysed and the membranes were isolated by flotation on a sucrose gradient. Of the resulting membrane fraction, 20 μg total protein was subjected to SDS-PAGE and Western blot. The YopB antibody recognizes a non-specific upper band that was used as a loading control, while the lower band is specific for YopB. The experiment was repeated three times and a representative experiment is shown. The image has been cropped to facilitate comparison between samples.

Previous studies have established a strong correlation between functional T3SS-mediated translocation and the ability to induce pore formation as measured by hemolytic activity in infected erythrocytes (Håkansson et al., 1996; Holmstrom et al., 1997; Neyt and Cornelis, 1999; Olsson et al., 2004; Ryndak et al., 2005). When the LcrV+1 and LcrV−1 strains were used to infect erythrocytes they were essentially unable to induce any significant hemolytic activity (Figure 1). In fact, the levels of hemolysis were found to be similar to that induced by Δ*lcrV*, Δ*yopB*, or Δ*yopD* mutants. Of note, the analysis was performed in a Δ*yopK* mutant background as the levels of both hemolysis and translocation, are known to be highly up-regulated in the absence of YopK (Holmstrom et al., 1997). These results indicate that the two LcrV+/−1 strains are unable to induce pore formation. Since functional LcrV is required for membrane insertion of YopB and YopD (Broz et al., 2007), we therefore wanted to establish if these mutants could still localize YopB and YopD to the cell membrane or not. To this end, membranes from infected erythrocytes were isolated by floatation on a sucrose gradient and subjected to SDS-PAGE and Western blot analysis using YopB and YopD antisera. By this approach, we were unable to detect YopB, and the levels of YopD were very low, in the membranes of cells infected with the two LcrV+/−1 strains both in parental background and in the Δ*yopK* mutant background (Figure 2). As expected (Thorslund et al., 2011), infection with the Δ*yopK* mutant resulted in increased levels of both YopB and YopD in the membranes (Figure 2). Control experiments verified that during infection the total levels of YopB and YopD were similar for all strains except for the Δ*lcrV* mutant, for which the expression of the T3SS encoding genes is known to be down-regulated (Pettersson et al., 1999) (Figure S2). Overall, the phenotype of the LcrV+/−1 strains was similar to a Δ*lcrV* mutant with respect to hemolytic activity and membrane insertion of YopB and YopD. Thus, the LcrV+/−1 strains evidently induced YopE mediated cytotoxicity, as reported previously (Bröms et al., 2007), without localizing YopB and YopD to the erythrocyte membrane.

### The LcrV+/−1 mutants translocate YopE and YopH despite lack of hemolytic activity

**Figure 3:**
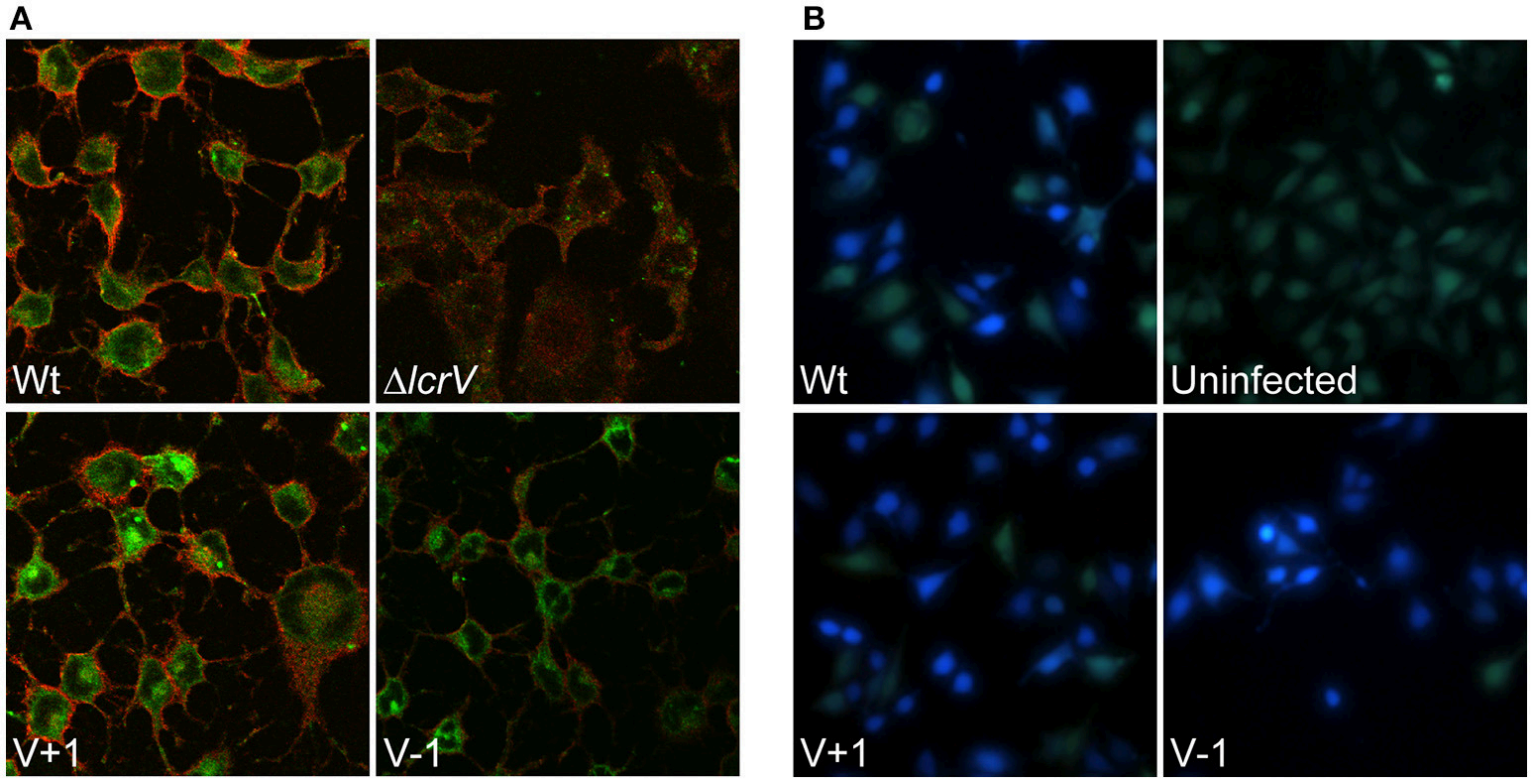
**Pore forming activity does not correlate to translocation ability. (A)** Intracellular localization of YopE in HeLa cells after 1.5 h infection was analyzed with immunostaining. The samples were viewed using laser scanning confocal microscopy and green staining corresponds to YopE, while the HeLa cell membranes are stained in red. **(B)** To analyze translocation of YopH, Hela cells were labeled with the FRET substrate CCF4-AM and infected with the indicated strains expressing YopH-Beta-lactamase fusion protein (YopH-Bla). Images were acquired in a live cell microscope using a longpass filter. Translocation of the Bla-fusion results in a shift of fluorescence from green to blue. Both experiments were repeated at least three times.

**Figure 4:**
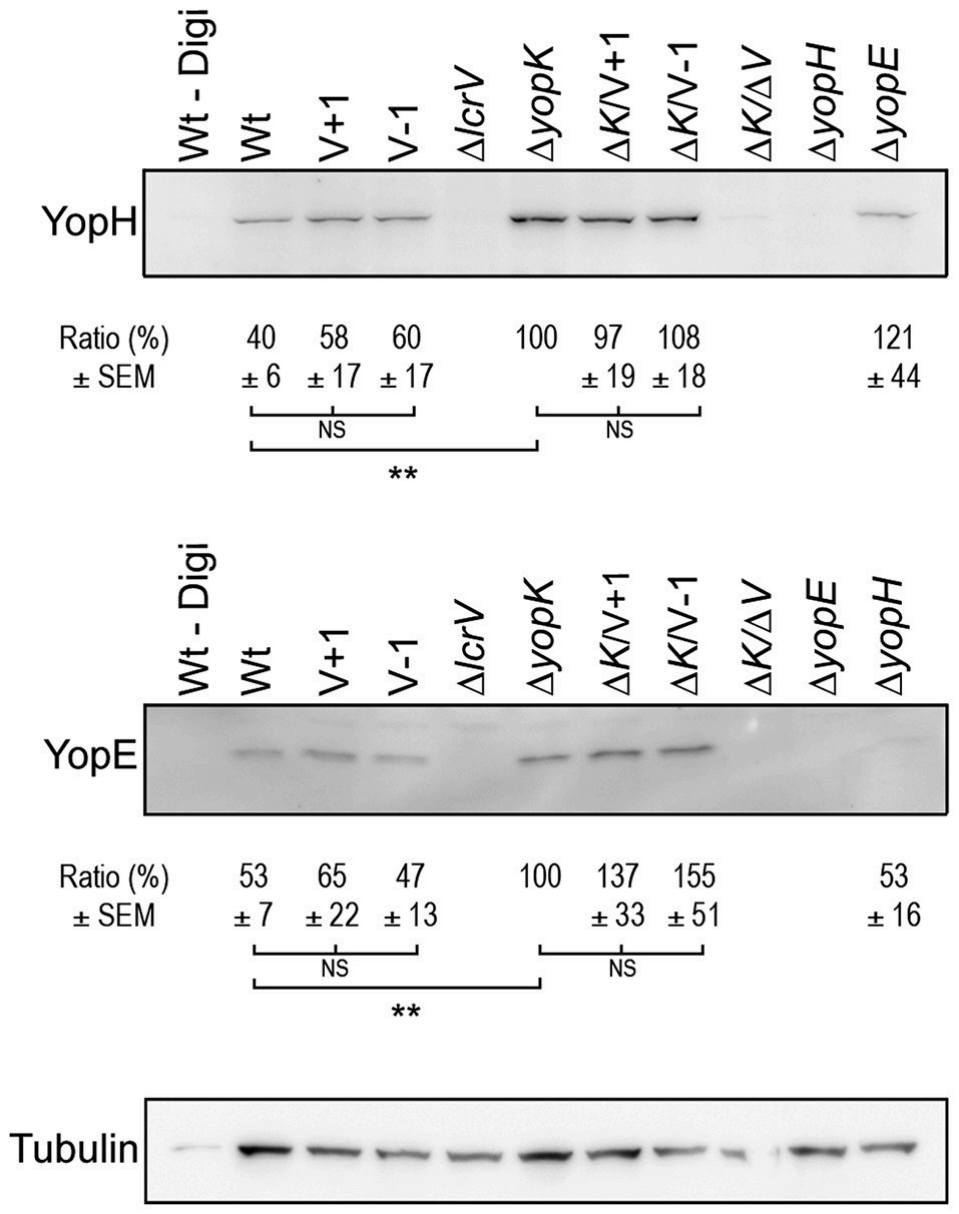
**Translocation levels are up-regulated even in the absence of pore formation**. Translocated YopH and YopE detected within HeLa cells after 1 h of infection (Δ*yopK*, Δ*K/V*+*1*, Δ*K/V–1* and Δ*K/*Δ*V*, Δ*yopE* and Δ*yopH*) or after 2 h (Wt, V+1, V−1, Δ*lcrV*). Extracellular proteins were removed by Proteinase K treatment after which the cells were lysed with 1% Digitonin. One sample (Wt–Digi) was left unlysed as a negative control. Detection of tubulin served as a loading control. The experiment was repeated at least three times and a representative experiment is shown. The signal intensity of the bands was quantified using the Multi Gauge-Image software (Fujifilm). The values for YopE and YopH were normalized to the loading control and the ratio ± SEM of translocated Yops relative to the Δ*yopK* mutant, is shown below each Western blot. The results were analyzed using the paired Student's *t*-test with the significance set at *p* ≤ 0.01^**^. NS, No significant difference.

As mentioned above, past studies have shown a strong correlation between lytic activity due to pore formation and the functional translocation of T3SS effectors (Håkansson et al., 1996; Holmstrom et al., 1997; Neyt and Cornelis, 1999; Olsson et al., 2004; Ryndak et al., 2005). Hence, it was important to verify that the LcrV+/−1 mutants that lacked hemolytic activity really had retained translocation ability. In our previous study we found that these mutants, similar to the wild type strain, induced rapid cytotoxicity in infected HeLa cells (Bröms et al., 2007). In this assay very low levels of translocated YopE are needed for visible cell rounding. Therefore, to detect any possible translocation deficiency, we repeated the assay using different MOIs. At a high MOI (40:1) the wild type strain induced full cytotoxicity within an hour of infection, while at a low MOI (2.5:1), full cytotoxicity was delayed until 3–4 h of infection. Using this approach it was clear that the LcrV+/−1 strains induced a cytotoxic response with similar kinetics as the wild type strain (data not shown), even at MOI 2.5. Since cytotoxic response is an indirect measurement of intracellular targeting of YopE, we decided to confirm more directly that YopE was translocated by the LcrV+/−1 strains. To this end, infected HeLa cells were fixed, permeabilized and incubated with rabbit-anti-YopE antisera followed by analysis using laser scanning confocal microscopy. YopE specific staining was detected in cells infected with the LcrV+/−1 mutants as well as in cells infected with the wild type, thereby confirming the ability of the mutants to translocate YopE (Figure 3A). Expectantly, no YopE specific staining was observed in cells infected with the Δ*lcrV* mutant. To confirm that this translocation was general and not just specific for YopE, we also monitored translocation of YopH using a beta-lactamase reporter system. HeLa cells were infected with *Yersinia* variants expressing a YopH-Bla fusion protein. Already after 40 min of infection, translocation of the fusion protein (as visualized by the blue fluorescence) was observed in a majority of cells infected with either the wild type or the LcrV+/−1 mutants (Figure 3B), once again confirming the general ability of the LcrV+/−1 mutants to translocate Yop effectors into the host cell. Quantification of the translocated effectors showed that the LcrV+/−1 mutants translocated YopE and YopH at wild type levels (Figure 4).

### Translocation by the LcrV+/−1 mutants is regulated by YopK

As previously mentioned, translocation is up-regulated in absence of YopK, which correlates with larger pores being formed (Holmstrom et al., 1997). This prompted us to investigate translocation of Yop effectors by the LcrV+1 and LcrV−1 strains in the Δ*yopK* mutant background, for which no pore formation was seen (Figure 1). To address this, HeLa cells infected with the different *Yersinia* strains were fractionated, using a well-established translocation assay (Nordfelth and Wolf-Watz, 2001). The cytosolic proteins were subjected to SDS-PAGE and Western blot using monospecific YopE and YopH antisera. As expected, the Δ*yopK* mutant translocated increased levels of both YopE and YopH compared to the isogenic wild type strain (Figure 4). The LcrV+1 and LcrV−1 strains also translocated increased levels of YopE and YopH in absence of YopK, at the same level as the Δ*yopK* mutant (Figure 4). No intracellular YopE and YopH could be detected in cells infected with the Δ*lcrV* mutant.

Since translocation of effectors occurred although little or no YopB and YopD could be detected in the erythrocyte membranes it was important to establish that effector translocation by the two LcrV+/−1 strains still required YopB and YopD. To address this, the LcrV+/−1 strains were also mutated for *yopB* and *yopD*. None of these strains were able to induce cytotoxicity of infected HeLa cells (data not shown) confirming that the effector translocation observed herein still required functional YopB and YopD.

It is puzzling that translocation in the LcrV+/−1 strains is regulated as in the wild type despite the lack of lytic activity. Contrasting with numerous previous studies, our results show that neither lytic activity nor translocator insertion in erythrocyte membranes have any correlation with levels of Yop effector translocation.

### The non-hemolytic LcrV+/−1 mutants insert YopD into HeLa cell membranes

**Figure 5:**
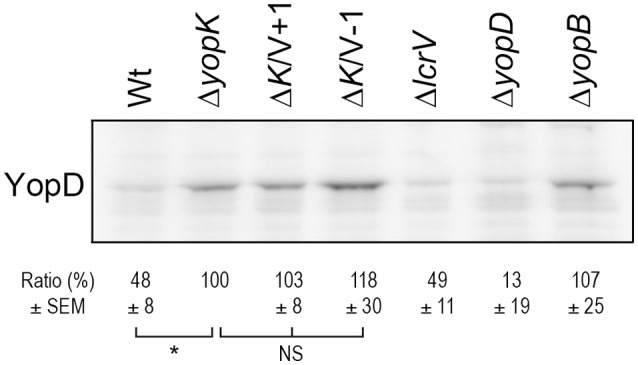
**The LcrV mutants promotes insertion of YopD into HeLa cell membranes**. HeLa cells were infected with *Y. pseudotuberculosis* for 2 h. The cells were lysed and the membranes isolated by a differential centrifugation technique. Of the resulting membrane fraction, equal amounts of total protein were subjected to SDS-PAGE and Western blot. The YopD antiserum recognized a non-specific band that served as a loading control (see original figure in Supplementary information, Figure S3). Membrane preparations of all samples were repeated at least twice. The signal intensity of the bands was quantified using the Multi Gauge-Image software (Fujifilm) and normalized to the unspecific band. The ratio ± SEM of YopD relative to the Δ*yopK* mutant, is shown below the Western blot. The results were analyzed using the paired Student's *t*-test with the significance set at *p* ≤ 0.05^*^. NS, No significant difference.

Given the observance of translocation in the absence of lytic activity, we decided to examine membrane localization of YopB and YopD in HeLa cells to be consistent with the experimental setup used in the translocation assays described above. We found that the levels of YopB were too low to be detected in cell membranes, regardless of the infecting strain (data not shown). The levels of YopD were very low, yet detectable by Western blot, in HeLa cells infected with the wild type strain (Figure 5). As expected, levels of membrane localized YopD were higher in cells infected with the Δ*yopK* mutant background and, most importantly, when the two LcrV+/−1 strains were analyzed in this background both mutants were found to insert similar levels of YopD into the membranes as the Δ*yopK* mutant strain (Figure 5). These findings are very important because above all they verify that using the erythrocyte model to study T3SS mediated pore formation and membrane insertion has limitations and can even give contradictory results that mislead conclusions regarding the T3SS mechanism. While the erythrocyte model is a convenient method, the relevance can be questioned, as erythrocytes are not a natural target cell of *Yersinia* during infection of an animal host.

### The LcrV N-terminus is involved in anti-phagocytosis and the intracellular targeting of YopH

With the exception of the experiments involving the erythrocyte infection model, the LcrV+/−1 strains displayed *in vitro* phenotypes that were very similar to the parental strain. Based on these observations, we anticipated that these mutants would be fully virulent. In order to examine this, we decided to use assays that could discriminate the impact of these mutations on *Yersinia* virulence.

**Figure 6:**
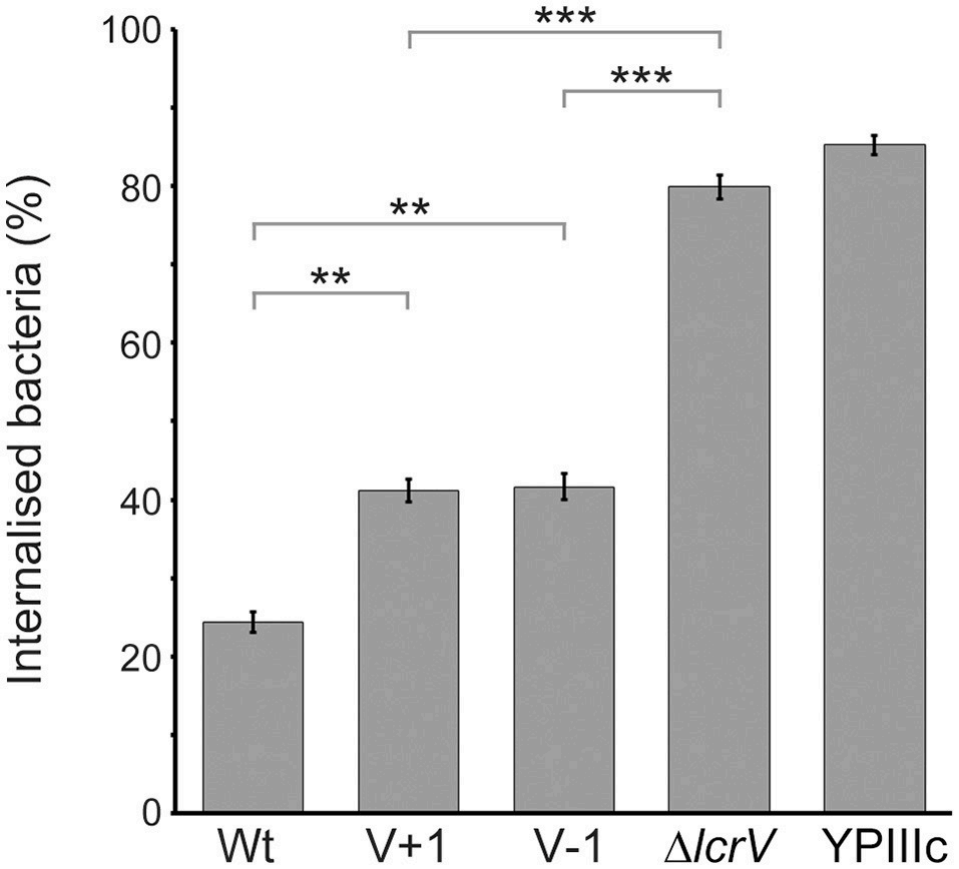
**The LcrV N-terminus is required for functional phagocytosis inhibition**. J774.a1 macrophages were infected with *Y. pseudotuberculosis* for 30 min after which the cells were fixed and extracellular as well as total bacteria were detected using a double staining technique. For each sample, the amount of total and extracellular bacteria of 50 infected cells was counted manually using a fluorescence microscope. The results presented are the mean value ± SEM of five independent experiments. The result of each experiment was analyzed using the Wilcoxon signed-rank test and the significance was set at *p* ≤ 0.01^**^ and *p* ≤ 0.001^***^.

One major virulence mechanism mediated by the T3SS of *Yersinia* is the ability to block phagocytosis by professional phagocytes (Rosqvist et al., 1988). To analyze if the LcrV+1 and LcrV−1 strains were affected in the ability to block phagocytosis we infected J774 macrophages with the different *Yersinia* strains for 30 min. The cells were then fixed and a double staining assay was performed to distinguish between extracellular and internalized bacteria. As expected, the wild type strain efficiently blocked phagocytosis with only 24% of infecting bacteria internalized (Figure 6). In contrast, 80% of the infecting Δ*lcrV* mutant and 85% of the virulence plasmid cured strain were phagocytosed (Figure 6). Interestingly, the LcrV+1 and LcrV−1 strains showed an intermediary phenotype where 41 and 42% of the bacteria were phagocytosed, respectively (Figure 6). Thus, even though the LcrV+/−1 strains were able to translocate both YopE and YopH to levels similar as the wild type strain, some critical aspect of this translocation process must be compromised in a manner that causes a significant reduction in phagocytosis inhibition.

**Figure 7:**
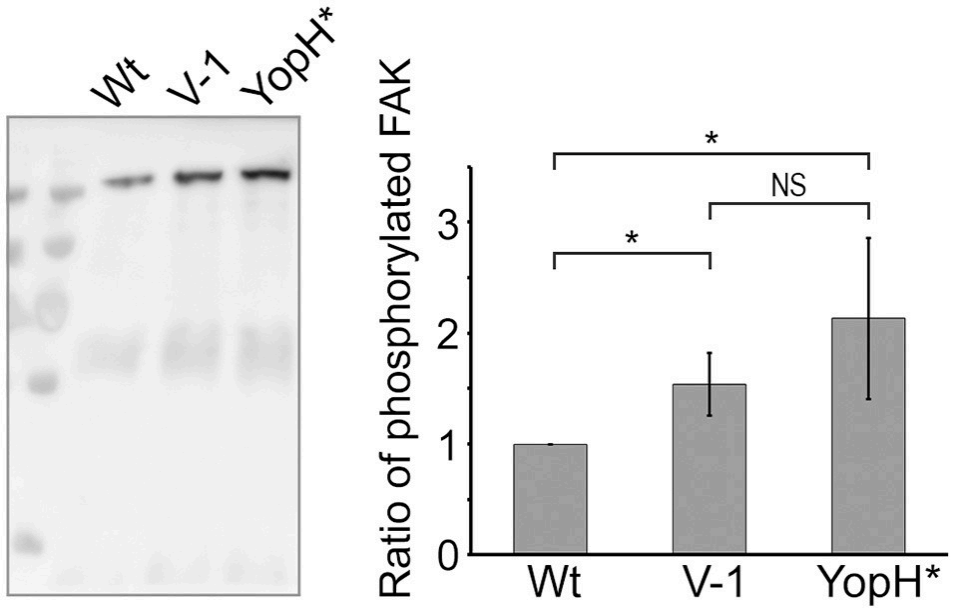
**Early targeting of translocated YopH is compromised in the LcrV frameshift mutants**. HeLa cells were infected with *Y. pseudotuberculosis* for 10 min with the indicated strains. The cells were lysed in RIPA buffer and the lysate was pre-cleared against mouse-IgG and then incubated with anti-FAK coated beads for 3 h. After washing, the bound material was eluted in a small volume of sample buffer and all material was subjected to SDS-PAGE and Western blot using the 4G10 antibody. Images were acquired in a LAS4000 image reader and the signal intensity was quantified using Multi Gauge-Image software (Fujifilm). The Western blot image is one representative experiment and the graph displays the ratio of average band intensity ± SD from quantification of three independent experiments. YopH^*^: YopH_C403A_. The results were analyzed using the paired Student's *t*-test with the significance set at *p* ≤ 0.05^*^. NS, No significant difference.

Previous work has established that early targeting of YopH to focal adhesion complexes is essential for phagocytosis inhibition (Andersson et al., 1996; Persson et al., 1997, 1999). One of the YopH targets is Focal Adhesion Kinase (FAK) and it was shown that the initial phosphorylation of FAK in response to *Yersinia* binding to the host cell is quickly reversed by the powerful phosphatase activity of YopH. By extension, this deactivation of FAK in turn impairs bacterial uptake (Persson et al., 1997). Hence, in order to establish if the N-terminal mutations in LcrV compromised early YopH targeting, we infected HeLa cells for 10 min and then immunoprecipitated FAK. The phosphorylation level of FAK was analyzed by Western blot, using phospotyrosine antibodies. In cells infected with the YopHC403A strain, FAK remained phosphorylated as this YopH variant is inactive and cannot dephosphorylate its target proteins. In contrast, in cells infected with the wild type strain, only low levels of phosphorylated FAK were detected (Figure 7). Curiously, the LcrV−1 strain also proved to be less efficient in blocking the phosphorylation of FAK compared to the wild type strain. Quantification of the levels of phosphorylated FAK from three independent experiments indicated a 2.13-fold reduction of phosphorylated FAK in cells infected with the wild type strain compared to the YopHC403A strain and a 1.54-fold reduction when compared to the LcrV−1 strain. Similar results were also observed with the LcrV+1 strain (data not shown). Thus, the LcrV+/−1 strains are defective in early targeting of YopH and this in turn impairs their ability to block phagocytosis (Figure 6). These are pertinent findings as they support the notion that LcrV both facilitates YopB/D localization to the host cell membrane and that the N-terminal region of LcrV mediates efficient early delivery of YopH to its molecular targets inside the infected host cell.

### Alterations of the LcrV N-terminal results in virulence attenuation *in vivo*

**Figure 8:**
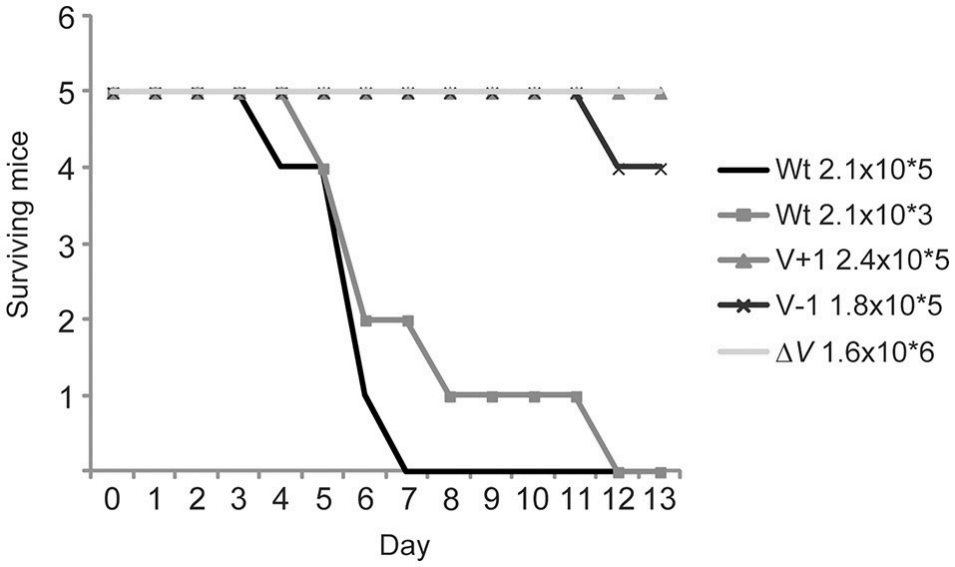
**The LcrV mutants are attenuated for virulence *in vivo***. C57/BL6 mice were infected intraperitoneally with *Y. pseudotuberculosis* at the indicated doses and signs of disease were monitored daily during 2 weeks. Mice that showed severe signs of disease, e.g., diarrhea, fussy fur and listlessness, were humanely sacrificed.

Since the LcrV+1 and LcrV–1 strains were impaired in anti-phagocytosis it was necessary to investigate if this correlated to attenuation in the *in vivo* mouse infection model. To this end, C57/BL6 mice were infected intraperitoneally with the various *Yersinia* strains. As expected the Δ*lcrV* mutant was completely attenuated, and all mice survived a high infection dose (1.6 × 10^6^ CFU) without displaying any signs of disease (fuzzy fur, diarrhea, weight loss, listlessness). Mice infected with the wild type strain showed disease symptoms within a few days even at the lowest dose used (2.1 × 10^3^ CFU) and all mice had succumbed to the infection by day 8 post infection. Strikingly, the LcrV+1 and LcrV−1 strains were essentially as attenuated as the Δ*lcrV* mutant. All but one mouse survived infection with the high dose of either LcrV+1 or LcrV−1 (Figure 8). Thus, the LcrV+1 and LcrV−1 strains were indeed highly attenuated for virulence, reflecting that a defect in early targeting of YopH has a dramatic impact on *in vivo* virulence.

## Discussion

The established role for the translocator LcrV is to facilitate insertion of YopB and YopD in host cell membranes to promote effector translocation (Matteï et al., 2011). Here, we show for the first time that the very N-terminal part of LcrV also has a specific role in early targeting of effectors to promote rapid blockage of phagocytosis in *Yersinia*. We have characterized two *lcrV* mutants that in contrast to the Δ*lcrV* mutant, expressed and secreted wild type levels of Yop effectors, but were unique in that they no longer could mediate efficient targeting of YopH to the focal adhesion sites. As the plasmid encoded T3SS is a key element in determining an extracellular pathogenic lifestyle of *Yersinia* the targeting defect of these mutants naturally had a dramatic effect on their *in vivo* virulence.

LcrV belongs to the class of hydrophilic translocators that locates to the tip of the needle complex of T3SS (Mueller et al., 2005). It has been shown that LcrV is required for translocation and promotes insertion of the two hydrophobic translocators into the host cell membrane (Pettersson et al., 1999; Broz et al., 2007). According to the injection model, the needle complex extends to contact the YopB/YopD induced pore and form a conduit for direct transport of the effectors from the cytosol of the bacterium into the host cell. Interestingly, studies from *P. aeruginosa* and *Shigella* indicate an active role of the tip proteins, PcrV and IpaD respectively, in sensing host cell contact (Lee et al., 2010; Schiavolin et al., 2013). In addition, it was recently proposed in *P. aeruginosa* that a complex of PcrV and the hydrophobic translocator PopD is involved in sensing host cell contact (Armentrout and Rietsch, 2016).

In human pathogenic *Yersinia* spp., LcrV has an additional regulatory role since Δ*lcrV* mutants are down-regulated for *yop* expression (Bergman et al., 1991; Skrzypek and Straley, 1995). To study the role of LcrV in translocation and virulence, it is therefore crucial to construct mutants with retained regulatory competence. Indeed, the two previously characterized *lcrV* mutants (LcrV+1 and LcrV−1) with completely altered amino acid sequences within the first 15 amino acids of the protein (Figure S1) maintained full control of both expression and secretion of translocators, including LcrV itself and effectors (Bröms et al., 2007). In addition, these mutants induced YopE mediated cytotoxicity on infected HeLa cells, indicating they retained an ability to translocate into infected cells (Bröms et al., 2007). When revisiting these mutants we observed that they were devoid of hemolytic activity and did not insert YopB or YopD into erythrocyte membrane, not even in a Δ*yopK* mutant background. We could however, confirm that both the LcrV+1 and the LcrV−1 strain translocated YopE and YopH at levels indistinguishable from the parental strain and we also verified that translocation was still dependent on YopB and YopD. Importantly, we also showed that translocation by the LcrV+/−1 strains was up-regulated in the absence of YopK. Collectively, these results show that translocation by the LcrV+/−1 strains is regulated in the same way as in the isogenic wild type strain, despite the lack of pore formation in erythrocytes. Remarkably, when we analyzed YopB/YopD membrane localization in nucleated cells we found that the LcrV+/−1 strains inserted similar levels of YopD in the HeLa cell membranes as the parental strain. Analyzing nucleated cells is far more challenging compared to erythrocytes and YopB levels proved to be below the detection limit in the Western blot analysis. Although we could not verify YopB insertion in HeLa cell membranes, we find it likely that the LcrV+/−1 strains also insert YopB along with YopD into the cell membrane. These results are important as they show that studies of pore formation in erythrocytes does not necessarily correlate to T3SS activity. This observation is reinforced by studies on YopD mutants that had a reduced hemolytic activity but maintained efficient and functional effector translocation (Costa et al., 2010). Numerous studies have used erythrocytes as the sole model system to study pore formation and its role in the T3SS mechanism of effector translocation across the host cell membrane. Those studies may need to be revisited to verify the findings in nucleated cells.

*Y. pseudotuberculosis* is an extracellular pathogen that replicates in the lymphatic tissues of the infected animal (Simonet et al., 1990). Therefore, the anti-phagocytic action mediated by the plasmid encoded T3SS, in *Yersinia*, is tremendously important (Rosqvist et al., 1988). Uptake of bacteria is triggered immediately after binding of the bacteria to the phagocytic cell. Hence, to block uptake, *Yersinia* must rapidly translocate the effectors upon cell contact. Critically, Andersson et al. have shown that YopH is rapidly translocated, reaching its eukaryotic targets within minutes after cell contact (Andersson et al., 1996; Persson et al., 1999). Here, we first established that the LcrV+1 and LcrV−1 strains translocated YopE and YopH as efficiently as the wild type strain when measured after 1–2 h of infection. However, this does not exclude the possibility that these *lcrV* mutations may have an effect on the early translocation after cell contact. Importantly, prolonged measurements of effector translocation may not always be a true reflection of the T3SS functional status. This is exemplified by the study of Thorslund and co-workers, where it was demonstrated that the ability to induce cytotoxicity did not correlate with the ability to block phagocytosis (Thorslund et al., 2011). Consistent with this, when we analyzed the LcrV+1 and LcrV−1 strains for phagocytosis inhibition, we found that they were significantly impaired in their ability to block phagocytosis. Together, these data demonstrate unequivocally that *in vitro* translocation levels after prolonged infection do not necessarily predict the outcome of an *in vivo* infection.

The YopE and YopH effectors mediate anti-phagocytic actions through different mechanisms. To block phagocytosis by macrophages, *Yersinia* relies heavily on YopH (Fahlgren et al., 2009), and further it has been shown that YopH is the effector that has the greatest impact on *in vivo* virulence (Logsdon and Mecsas, 2003). YopH is translocated immediately after cell contact and is actively targeted to the focal adhesion sites by the T3SS (Thorslund et al., 2011). In line with our results on phagocytosis inhibition, the early targeting of YopH to FAK was impaired in the LcrV+/−1 mutants, which suggests that the inability of the *lcrV* mutants to block phagocytosis is due to a defect in the intracellular targeting of translocated YopH. Thus, despite there being no disparity in the levels of translocated YopH accumulated after prolonged infection, by wild type and mutant strains, the early targeting of YopH was dramatically affected in our mutants. As evidenced from this work, future studies must recognize the importance of analyzing translocation at the early time points of infection. Using our *in vivo* infection model, LcrV+1 and LcrV−1 strains were almost fully attenuated for virulence and equivalent to the non-translocating Δ*lcrV* mutant. Therefore, it seems that the consequence of inefficient targeting of YopH is as severe as not being able to translocate at all. In agreement with this, Persson and co-workers have previously shown that a YopH variant, which failed to associate YopH with the focal adhesion sites was strongly reduced in phagocytosis inhibition and was also attenuated for virulence *in vivo* (Persson et al., 1999). Interestingly, our results show that these *lcrV* mutations result in a comparable phenotype.

Based on the results presented here, we propose that LcrV, in addition to promoting membrane insertion of YopB and YopD, also has a role in the early targeting of YopH in the eukaryotic cell. The *LcrV*+*/*−*1* mutations are positioned within a postulated unstructured region that is somehow recognized for secretion. This region is not part of the solved LcrV crystal structure (Derewenda et al., 2004; Chaudhury et al., 2013), and this makes it difficult to predict what impact the mutations could have on the overall structure/function of LcrV. However, it is feasible that it could have some impact on the LcrV/YopD interaction, which is known to be mediated primarily by the N-terminal domain immediately downstream of the *LcrV*+*/*−*1* mutations (Broz et al., 2007; Armentrout and Rietsch, 2016). This could in turn have an impact on the cell contact-mediated induction and effector translocation in line with what was recently proposed in *Pseudomonas* (Armentrout and Rietsch, 2016). Considering the proposed model where YopK directs YopH intracellularly by bridging RACK1 to the translocon, through YopD (Thorslund et al., 2011; Dewoody et al., 2013), it is possible that LcrV via its interaction with YopD impacts on the YopH targeting efficiency.

It is very intriguing that the LcrV+/−1 mutants, which initially appeared to possess very subtle phenotypes using established assays to study T3SS function, in fact turned out to have a major impact on phagocytosis inhibition and *in vivo* virulence. This highlights that for an extracellular pathogen like *Yersinia*, it is the early events directly after cell contact that are crucial for the outcome of the infection. This is very important since most studies of T3SS secretion and effector translocation are routinely performed after a few hours of host cell infection. One obvious reason for this is that protein levels are only readily detectable at these time points. Yet, our findings clearly signal that for future research on the molecular mechanism of T3SSs it is critical to assess the early events after bacteria host cell contact. We also conclude that using erythrocytes to study pore formation in relation to T3SS mechanism and function is not recommended if future research in the field is to yield meaningful data.

## Materials and methods

### Growth conditions

The bacterial strains and plasmids used in this study are presented in Table 1. Bacteria were routinely grown at 26°C in Luria Bertani (LB) agar or LB broth supplemented with 1 mM CaCl_2_ and 75 mM NaCl. For induction of the T3SS, bacteria were grown at 37°C in secretion permissive medium (5 mM EGTA and 20 mM MgCl_2_) (LB–Ca^2+^) unless otherwise stated. Where appropriate the following antibiotics were added to the final concentrations; kanamycin 30 μg/ml, chloramphenicol 25 μg/ml.

**Table 1:** **Strains and plasmids used in this study**.

**Strains and Plasmids**	**Relevant genotype**	**Reference**
**Strain**		
*** E. coli***		
S17-1λ*pir*	*recA, thi, pro, hsdR^−^M^+^*, Sm^R^, <RP4:2-Tc:Mu:Ku:Tn7>Tp^R^	Simon et al., 1983
*** Y. pseudotuberculosis***		
YPIII_C_	Plasmid cured strain	Bölin et al., 1982
YPIII pIB102	*yadA*; Km^r^ (wild type)	Bölin and Wolf-Watz, 1984
YPIII pIB19	pIB102: *lcrV* full-length in frame deletion; Km^r^	Pettersson et al., 1999
YPIII pIB155	pIB102: *yopK* full-length in frame deletion; Km^r^	Holmstrom et al., 1997
YPIII pIB621	pIB102: *yopD* full-length in frame deletion; Km^r^	Olsson et al., 2004
YPIII pIB615	pIB102: *yopB* full-length in frame deletion; Km^r^	Bröms et al., 2003
YPIII pIB30	pIB102: *yopH* full-length in frame deletion; Km^r^	This study
YPIII pIB526	pIB102: *yopE* full-length in frame deletion; Km^r^	Aili et al., 2008
YPIII pIB15519	pIB155: *lcrV* full-length in frame deletion; Km^r^	Holmstrom et al., 2001
YPIII pIB10201	pIB102: *lcrV* +1 frameshift mutation in codons 4–13; Km^r^ (V+1)	Bröms et al., 2007
YPIII pIB10202	pIB102: *lcrV* −1 frameshift mutation in codons 2–15; Km^r^ (V−1)	Bröms et al., 2007
YPIII pIB1550201	pIB155: *lcrV* +1; Km^r^	This study
YPIII pIB1550202	pIB155: *lcrV* −1; Km^r^	This study
YPIII pIB6210201	pIB621: *lcrV* +1; Km^r^	This study
YPIII pIB6210202	pIB621: *lcrV* −1; Km^r^	This study
YPIII pIB6150201	pIB615: *lcrV* +1; Km^r^	This study
YPIII pIB6150202	pIB615: *lcrV* −1; Km^r^	This study
YPIII pIB102YopH-Bla	Wild type, expressing YopH_FL_-Bla	This study
YPIII pIB10201YopH-Bla	LcrV +1, expressing YopH_FL_-Bla	This study
YPIII pIB10202YopH-Bla	LcrV −1, expressing YopH_FL_-Bla	This study
YPIII pIB102 pYopH_C403A_	Wild type, expressing catalytically inactive YopH	This study
**Plasmid**		
pJEB368	pDM4 *lcrV* + 1 frameshift encompassing codon 4–13	Bröms et al., 2007
pJEB369	pDM4 *lcrV* −1 frameshift encompassing codon 2–15	Bröms et al., 2007
pMF024	pDM4 *yopD* full-length in frame deletion	Francis and Wolf-Watz, 1998
pMF463	pDM4 *yopB* full-length in frame deletion	Bröms et al., 2003
pDM4-ΔyopH	pDM4 *yopH* full-length in frame deletion	Westermark et al., 2014
pNQ-YopH_FL_-Bla	pNQ705 with YopH_1−468_-Bla_24−286_ fusion	This study
pYopH_C403A_	Point mutation in the catalytic site of YopH, cloned under its own promoter	Persson et al., 1997

HeLa cells were cultivated in Minimum essential medium (MEM) (Sigma) supplemented with 10% fetal bovine serum (FBS) (Sigma), 2 mM glutamine, 0.035% sodium bicarbonate and 100 IU penicillin. J774.1 cells were cultivated in Dulbecco's modified eagle medium (DMEM glutaMAX) (Gibco) supplemented with 10% FBS and 100 IU penicillin. The cells were kept at 37°C with 5% CO_2_. For infection assays cells were seeded in plates 1 day before experiment and 0.5 hour before infection the cells were washed and incubated in non-supplemented MEM or DMEM.

### Construction of mutants

*E. coli* S17-1 λ*pir* was used to conjugate the suicide plasmids pJEB368 and pJEB369 into YPIII/pIB155 (Δ*yopK*) to generate Δ*yopK*/LcrV+1 and Δ*yopK*/LcrV−1 mutants. Δ*yopB* and Δ*yopD* double mutants were constructed by conjugating the suicide plasmids pMF024 and pMF463 into YPIIIpIB10201 (LcrV+1) and YPIIIpIB10202 (LcrV−1). The YopH-Bla fusion was constructed by overlap PCR using the primers listed in Table S1, yielding a DNA fragment corresponding to codons 6–468 of YopH fused to beta-lactamase codons 24–286 (Akopyan et al., 2011). The PCR fragment was cloned into the pcr2.1 TOPO vector (Invitrogen) for amplification, and then sub-cloned into the pNQ705 suicide vector (Milton et al., 1992). S17-1 λpir was used as the donor strain in conjugation with *Yersinia pseudotuberculosis* wild type (YPIII pIB102), LcrV+1 (YPIII pIB10201) and LcrV−1 (YPIII pIB10202) strains and single recombination of the pNQ-YopH_FL_-Bla inactivated the wild type copy of YopH.

### Lytic activity and membrane isolation from erythrocytes

Sheep erythrocytes were washed and resuspended in LB–Ca^2+^ Supplemented with 75 mM NaCl and Complete Mini protease inhibitor cocktail (PIC) (Roche). A total of 5 × 10^9^ induced *Yersinia* bacteria were harvested and resuspended in 5 ml erythrocytes suspension (10^11^ cells) and centrifuged to create contact between bacteria and cells before incubation at 37°C. Samples were repeatedly resuspended and centrifuged during infection to increase contact between bacteria and cells. After 2 h 100 μL samples were taken and mixed 1:1 with PBS and centrifuged at 3500 g, 5 min, after which 100 μl of the supernatant was transferred to a flat bottom plate and the absorbance at 570 nm was measured to determine the hemolysis.

To analyze total levels of YopB and YopD during infection, 500 mL samples were collected, mixed 1:1 with MQ H_2_O and vortexed to lyse the cells. The samples were treated with DNAseI (Thermo Fisher Scientific) according to the manufactures instructions. The samples were centrifuged at 12,000 g, 5 min and the supernatants were collected and subjected to SDS-PAGE and Western blot using an antisera which recognizes both YopB and YopD (ASTI).

To isolate the erythrocytes membranes, 35 ml ice-cold lysis buffer (5 mM Tris-HCl, PIC, pH 7.5) was added to the remaining reactions followed by shaking and incubation on ice 10 min, then 4.5 ml 10X TBS (200 mM Tris-HCl, 1.5 M NaCl, pH 7.5) was added to restore the pH and salt concentration and the samples were centrifuged at 3500 g, 20 min. The supernatants were transferred to thick-wall centrifugation tubes (Beckman) and spun at 100,000 g for 2 h at 4°C. The pellet was resuspended in 50% sucrose in TBS (20 mM Tris-HCl, 150 mM NaCl, PIC, pH 7.5) and sonicated. A step-wise gradient was established in ultra-clear centrifugation tubes by adding the resuspended sample on top of 2 ml 65% sucrose-TBS and overlay with 15 ml 44% sucrose-TBS followed by 5 ml 25% sucrose-TBS. Gradients were centrifuged at 15,000 g for 16 h at 4°C using a Sw-28 rotor (Beckman). The membranes were isolated from the 25/44% interface and washed with TBS before resuspension in 150 μl TBS. The total protein concentration was estimated in a NanoDrop spectrophotometer at A_280_ and 20 μg was loaded on a 12% SDS-PAGE and analyzed by Western blot using YopD polyclonal antisera and YopB monoclonal antibody.

### Intracellular localization of YopE and YopH

A total of 10^5^ HeLa cells were infected with induced *Yersinia* strains at MOI ranging from 2.5:1 to 40:1 and the level of cytotoxicity was observed in an inverted light microscope, repeatedly during up to 4 h post infection. For immunostaining a MOI of 25:1 was used. The cells were washed and fixed in 2% paraformaldehyde after 1.5 h infection. The cell membranes were stained with TexasRed conjugated wheat germ agglutinin (Molecular Probes, Invitrogen) before they were permeabilised in TSB (0.5% Triton X-100 in a buffer consisting of 4% PEG 6000, 1 mM EGTA and 100 mM Pipes pH 6.8). Intracellular YopE was detected using an affinity purified polyclonal YopE antibody followed by an Alexa488-conjugated secondary antibody (Molecular probes, Invitrogen). The nucleus and bacteria were stained with DAPI. Images were taken with laser scanning confocal microscopy (Leica).

YopH translocation was analyzed using a beta-lactamase reporter system (Charpentier and Oswald, 2004; Marketon et al., 2005) where full length YopH was fused to beta-lactamase. HeLa cells were seeded in 35 mm glass bottom dishes (MatTek) 1 day before the experiment. Prior to infection the cells were labeled with the FRET substrate CCF4-AM (Invitrogen) according to the manufactures instructions. Induced *Yersinia* strains were added to the cells at a MOI of 50:1 and incubated 40 min at room temperature. Images were taken with a live cell microscope (Nikon Eclipse Ti-E), equipped with a true color camera, using a long pass filter to detect the two wave length of the FRET substrate.

### Proteinase K protection assay

The experiment was performed essentially as described before (Nordfelth and Wolf-Watz, 2001). In short, 5 × 10^6^ HeLa cells were infected with induced *Yersinia* strains at a MOI of 50:1 in the presence of Cytochalasin D (0.5 μg/ml). After infection the cells were washed in PBS and treated with 0.5 mg/ml Proteinase K (Roche Diagnostics, Gmbh) for 1 min. The cells were aspirated and incubated 20 min at room temperature, after which, 4 mM PMSF was added to inactivate remaining Proteinase K. The cells were lysed with 0.4% Digitonin in a total volume of 1 ml. Cell debris and bacteria were pelleted by centrifugation at 15,000 g, 10 min, 4°C. The supernatant was separated on a 12% SDS-PAGE and analyzed by Western blot using affinity purified antisera against YopE and YopH or monoclonal Tubulin antibody (Sigma Aldrich). The protein levels were quantified by use of the LAS4000 image reader and Multi Gauge-Image software (Fujifilm). The levels of YopE and YopH were normalized against Tubulin levels. The results were analyzed using the paired Student's *t*-test with the significance set at *p* ≤ 0.05^*^, *p* ≤ 0.01^**^ and *p* ≤ 0.001^***^.

### Isolation of plasma membrane from HeLa cells

A total of 10^7^ HeLa cells were infected in duplicates with *Yersinia* strains at a MOI of 50:1 in 15 cm dishes. After 1 h at 37°C the cells were put on ice for 5 min and washed twice with ice cold PBS. The cells were scraped off in 5 mL ice-cold PBS and suspension from the duplicate dishes were pooled in a centrifugation tube. The cells were pelleted at 290 g, 5 min, resuspended in 1 mM EGTA, 1 mM MgCl_2_, 1 mM DTT, and incubated on ice for 8 min after which the cells were pelleted again at 290 g, 5 min. The cells were resuspended in 0.3 mL 250 mM sucrose, 3 mM Imidazole and Complete Mini protease inhibitor cocktail (Roche) (total volume ~0.4 mL) and lysed by passing 40 times through a 23 G needle. The lysate was centrifuged at 900 g, 5 min and the supernatant was collected. The pellet was washed with 1 mL sucrose buffer and spun again at 900 g and the supernatant was pooled to the previous one and the samples were spun at 14000 g, 5 min. The resulting supernatant was centrifuged at 125000 g for 1 h using a Ti70.1 rotor (Beckman). The pellet containing the cell membranes was resuspended in 100 μL 2X SDS loading buffer. Equal amounts of protein were loaded on a 12% SDS-gel and analyzed with Western blot using YopD polyclonal antisera. The levels of YopD were quantified using the Multi Gauge-Image software (Fujifilm) and normalized to the unspecific band. The results were analyzed using the paired Student's *t*-test with the significance set at *p* ≤ 0.05^*^, *p* ≤ 0.01^**^ and *p* ≤ 0.001^***^. NS, No significant difference.

### Phagocytosis inhibition assay

A total of 10^5^ J774a.1 cells were infected with *Yersinia* strains at a MOI of 20:1. After 30 min infection unattached bacteria were washed away with PBS and the cells were fixed in 0.4% paraformaldehyde. Extracellular bacteria were stained using *Yersinia* antisera followed by Alexa568-conjugated antibody (Molecular Probes, Invitrogen). The cells were permeabilised with 0.5% Triton X-100 and both extra- and intracellular bacteria were stained with *Yersinia* antisera followed by Alexa488-conjugated antibody (Molecular Probes, Invitrogen). Samples were viewed in a fluorescence microscope and the total amount of cell associated bacteria and extracellular bacteria were counted manually. The results were analyzed using the Wilcoxon signed-rank test. Each experiment was analyzed separately and significance was set at *p* ≤ 0.05^*^, *p* ≤ 0.01^**^ and *p* ≤ 0.001^***^.

### Immunoprecipitation

A total of 4 × 10^6^ HeLa cells were infected with *Yersinia* strains at a MOI of 100:1 in a small volume to create instant contact between bacteria and cells. The infection was terminated at different time points by washing two times with ice-cold PBS + 0.1 mM Na_3_VO_4_. The cells were lysed in ice-cold precipitation buffer [50 mM Tris, 150 mM NaCl, 1 mM EGTA, 1% Nonidet NP-40, 0.25% sodium deoxycholate, 1 mM Na_3_VO_4_ and Complete Mini protease inhibitor cocktail (Roche Diagnostics, Gmbh)] at 4°C for 20 min after which the cells were scraped off and centrifuged at 16000 g for 10 min. The lysate was pre-incubated with mouse IgG coated protein G Sepharose beads (4 Fast Flow, Amersham Biosciences, Sweden) at 4°C for 1 h. The beads were spun down and the pre cleared lysate was incubated with anti-FAK (clone 2A7, Upstate Biotechnology, Lake Placid, NY) coated proteinG sepharose beads at 4°C for 3 h. The beads were washed two times in precipitation buffer and the bound material was eluted in a small volume of 2X SDS loading buffer at 95°C for 5 min. Samples were subjected to SDS-PAGE and the amount of phosphorylated FAK was analyzed by Western Blot using a phosphotyrosine antibody (Clone 4G10) (Millipore). Images were acquired in a LAS4000 image reader and the signal intensity was quantified using Multi Gauge-Image software (Fujifilm). The results were analyzed using the paired Student's *t*-test with the significance set at *p* ≤ 0.05^*^, *p* ≤ 0.01^**^ and *p* ≤ 0.001^***^. NS, No significant difference.

### Mouse infection model

Bacteria were grown overnight in unsupplemented LB at room temperature and harvested and diluted in PBS to final concentrations ranging from 10^4^to 10^6^ bacteria/ml for the wild type and LcrV frameshift mutants. The attenuated Δ*lcrV* mutant was used as a negative control at concentrations 10^5^–10^7^ bacteria/ml. Five C57BL/6 mice were infected intraperitoneally with 100 μl for each strain and dilution. Bacteria were plated on LA plates in parallel to determine the exact infection dose. The experiments were conducted in accordance with the ethical permission and all mice were monitored for symptoms twice daily and mice showing severe symptoms were sacrificed. Based on our previous experience mice with severe symptoms normally succumb to the infection within 24 h. The animal experiment was approved by the local animal ethics committee at Umea University (Dnr A144-12).

## Author contributions

SE, JB, TE, MF, MSF and ÅF conceived and designed the experiments. SE, JB, and ÅF performed the experiments. SE and JB analyzed the data. SE, MF, MSF and ÅF wrote the manuscript.

## Funding

This work was supported by grants from the Swedish Research Council, 2011-3439, and the Swedish Foundation for Strategic Research, SB12-0022.

### Conflict of interest statement

The authors declare that the research was conducted in the absence of any commercial or financial relationships that could be construed as a potential conflict of interest.

## References

[B1] Aili M. Isaksson E. L. Carlsson S. E. Wolf-Watz H. Rosqvist R. Francis M. S. (2008). Regulation of Yersinia Yop-effector delivery by translocated YopE. Int. J. Med. Microbiol. 298, 183–192. 10.1016/j.ijmm.2007.04.007 17597003

[B2] Akopyan K. Edgren T. Wang-Edgren H. Rosqvist R. Fahlgren A. Wolf-Watz H.. (2011). Translocation of surface-localized effectors in type III secretion. Proc. Natl. Acad. Sci. U.S.A. 108, 1639–1644. 10.1073/pnas.1013888108 21220342 PMC3029700

[B3] Andersson K. Carballeira N. Magnusson K.-E. Persson C. Stendahl O. Wolf-Watz H.. (1996). YopH of *Yersinia pseudotuberculosis* interrupts early phosphotyrosine signalling associated with phagocytosis. Mol. Microbiol. 20, 1057–1069. 10.1111/j.1365-2958.1996.tb02546.x 8809758

[B4] Andersson K. Magnusson K. E. Majeed M. Stendahl O. Fällman M. (1999). *Yersinia pseudotuberculosis*-induced calcium signaling in neutrophils is blocked by the virulence effector YopH. *Infect*. Immun. 67, 2567–2574. 10.1128/iai.67.5.2567-2574.1999 PMC116005 10225922

[B5] Armentrout E. I. Rietsch A. (2016). The type III secretion translocation pore senses host cell contact. PLoS Pathog. 12:e1005530. 10.1371/journal.ppat.1005530 27022930 PMC4811590

[B6] Bergman T. Håkansson S. Forsberg A. Norlander L. Macellaro A. Bäckman A.. (1991). Analysis of the V antigen lcrGVH-yopBD operon of *Yersinia pseudotuberculosis*: evidence for a regulatory role of LcrH and LcrV. J. Bacteriol. 173, 1607–1616. 10.1128/jb.173.5.1607-1616.1991 1705541 PMC207309

[B7] Bölin I. Norlander L. Wolf-Watz H. (1982). Temperature-inducible outer membrane protein of *Yersinia pseudotuberculosis* and *Yersinia enterocolitica* is associated with the virulence plasmid. *Infect*. Immun. 37, 506–512. 10.1128/iai.37.2.506-512.1982 PMC347563 6749681

[B8] Bölin I. Wolf-Watz H. (1984). Molecular cloning of the temperature-inducible outer membrane protein 1 of *Yersinia pseudotuberculosis. Infect*. Immun. 43, 72–78. 10.1128/iai.43.1.72-78.1984 PMC263390 6317574

[B9] Bröms J. E. Francis M. S. Forsberg A. (2007). Diminished LcrV secretion attenuates *Yersinia pseudotuberculosis* virulence. J. Bacteriol. 189, 8417–8429. 10.1128/JB.00936-07 17873031 PMC2168923

[B10] Bröms J. E. Sundin C. Francis M. S. Forsberg A. (2003). Comparative analysis of type III effector translocation by *Yersinia pseudotuberculosis* expressing native LcrV or PcrV from *Pseudomonas aeruginosa*. J. Infect. Dis. 188, 239–249. 10.1086/376452 12854079

[B11] Broz P. Mueller C. A. Müller S. A. Philippsen A. Sorg I. Engel A.. (2007). Function and molecular architecture of the Yersinia injectisome tip complex. Mol. Microbiol. 65, 1311–1320. 10.1111/j.1365-2958.2007.05871.x 17697254

[B12] Charpentier X. Oswald E. (2004). Identification of the secretion and translocation domain of the enteropathogenic and enterohemorrhagic *Escherichia coli* effector Cif, using TEM-1 β-lactamase as a new fluorescence-based reporter. J. Bacteriol. 186, 5486–5495. 10.1128/JB.186.16.5486-5495.2004 15292151 PMC490934

[B13] Chaudhury S. Battaile K. P. Lovell S. Plano G. V. De Guzman R. N. (2013). Structure of the *Yersinia pestis* tip protein LcrV refined to 1.65 Å resolution. Acta Crystallogr. Sect. F Struct. Biol. Cryst. Commun. 69, 477–481. 10.1107/S1744309113008579 23695558 PMC3660882

[B14] Costa T. R. Edqvist P. J. Bröms J. E. Ahlund M. K. Forsberg A. Francis M. S. (2010). YopD self-assembly and binding to LcrV facilitate type III secretion activity by *Yersinia pseudotuberculosis*. J. Biol. Chem. 285, 25269–25284. 10.1074/jbc.M110.144311 20525687 PMC2919090

[B15] Derewenda U. Mateja A. Devedjiev Y. Routzahn K. M. Evdokimov A. G. Derewenda Z. S.. (2004). The structure of *Yersinia pestis* V-antigen, an essential virulence factor and mediator of immunity against plague. Structure 12, 301–306. 10.1016/j.str.2004.01.010 14962390

[B16] Dewoody R. Merritt P. M. Marketon M. M. (2013). YopK controls both rate and fidelity of Yop translocation. Mol. Microbiol. 87, 301–317. 10.1111/mmi.12099 23205707 PMC3545096

[B17] Dohlich K. Zumsteg A. B. Goosmann C. Kolbe M. (2014). A substrate-fusion protein is trapped inside the type III secretion system channel in *Shigella flexneri*. PLoS Pathog. 10:e1003881. 10.1371/journal.ppat.1003881 24453973 PMC3894212

[B18] Elsinghorst E. A. Baron L. S. Kopecko D. J. (1989). Penetration of human intestinal epithelial cells by salmonella: molecular cloning and expression of salmonella typhi invasion determinants in *Escherichia coli*. Proc. Natl. Acad. Sci. U.S.A. 86, 5173–5177. 10.1073/pnas.86.13.5173 2662196 PMC297580

[B19] Fahlgren A. Westermark L. Akopyan K. Fällman M. (2009). Cell type-specific effects of *Yersinia pseudotuberculosis* virulence effectors. Cell. Microbiol. 11, 1750–1767. 10.1111/j.1462-5822.2009.01365.x 19681909

[B20] Finlay B. B. Starnbach M. N. Francis C. L. Stacker B. A. Chatfield S. Dougan G.. (1988). Identification and characterization of TnphoA mutants of salmonella that are unable to pass through a polarized MDCK epithelial cell monolayer. Mol. Microbiol. 2, 757–766. 10.1111/j.1365-2958.1988.tb00087.x 2850443

[B21] Francis M. S. Wolf-Watz H. (1998). YopD of *Yersinia pseudotuberculosis* is translocated into the cytosol of HeLa epithelial cells: evidence of a structural domain necessary for translocation. Mol. Microbiol. 29, 799–813. 10.1046/j.1365-2958.1998.00973.x 9723919

[B22] Frithz-Lindsten E. Du Y. Rosqvist R. Forsberg Å. (1997). Intracellular targeting of exoenzyme S of *Pseudomonas aeruginosa* via type III-dependent translocation induces phagocytosis resistance, cytotoxicity and disruption of actin microfilaments. Mol. Microbiol. 25, 1125–1139. 10.1046/j.1365-2958.1997.5411905.x 9350868

[B23] Frithz-Lindsten E. Holmström A. Jacobsson L. Soltani M. Olsson J. Rosqvist R.. (1998). Functional conservation of the effector protein translocators PopB/YopB and PopD/YopD of *Pseudomonas aeruginosa* and *Yersinia pseudotuberculosis*. Mol. Microbiol. 29, 1155–1165. 10.1046/j.1365-2958.1998.00994.x 9767584

[B24] Galán J. E. Wolf-Watz H. (2006). Protein delivery into eukaryotic cells by type III secretion machines. Nature 444, 567–573. 10.1038/nature05272 17136086

[B25] Håkansson S. Schesser K. Persson C. Galyov E. E. Rosqvist R. Homblé F.. (1996). The YopB protein of *Yersinia pseudotuberculosis* is essential for the translocation of Yop effector proteins across the target cell plasma membrane and displays a contact-dependent membrane disrupting activity. EMBO J. 15, 5812–5823. 8918459 PMC452329

[B26] Hanski C. Kutschka U. Schmoranzer H. P. Naumann M. Stallmach A. Hahn H.. (1989). Immunohistochemical and electron microscopic study of interaction of *Yersinia enterocolitica* serotype O8 with intestinal mucosa during experimental enteritis. Infect. Immun. 57, 673–678. 2917779 10.1128/iai.57.3.673-678.1989 PMC313160

[B27] Holmstrom A. Olsson J. Cherepanov P. Maier E. Nordfelth R. Pettersson J.. (2001). LcrV is a channel size-determining component of the Yop effector translocon of Yersinia. Mol. Microbiol. 39, 620–632. 10.1046/j.1365-2958.2001.02259.x 11169103

[B28] Holmstrom A. Pettersson J. Rosqvist R. Håkansson S. Tafazoli F. Fällman M.. (1997). YopK of *Yersinia pseudotuberculosis* controls translocation of Yop effectors across the eukaryotic cell membrane. Mol. Microbiol. 24, 73–91. 10.1046/j.1365-2958.1997.3211681.x 9140967

[B29] Lee P.-C. Stopford C. M. Svenson A. G. Rietsch A. (2010). Control of effector export by the *Pseudomonas aeruginosa* type III secretion proteins PcrG and PcrV. Mol. Microbiol. 75, 924–941. 10.1111/j.1365-2958.2009.07027.x 20487288 PMC3124366

[B30] Logsdon L. K. Mecsas J. (2003). Requirement of the *Yersinia pseudotuberculosis* effectors YopH and YopE in colonization and persistence in intestinal and lymph tissues. Infect. Immun. 71, 4595–4607. 10.1128/IAI.71.8.4595-4607.2003 12874339 PMC166012

[B31] Marketon M. M. DePaolo R. W. DeBord K. L. Jabri B. Schneewind O. (2005). Plague bacteria target immune cells during infection. Science 309, 1739–1741. 10.1126/science.1114580 16051750 PMC3210820

[B32] Matteï P.-J. Faudry E. Job V. Izoré T. Attree I. Dessen A. (2011). Membrane targeting and pore formation by the type III secretion system translocon. FEBS J. 278, 414–426. 10.1111/j.1742-4658.2010.07974.x 21182592

[B33] Milton D. L. Norqvist A. Wolf-Watz H. (1992). Cloning of a metalloprotease gene involved in the virulence mechanism of *Vibrio anguillarum*. J. Bacteriol. 174, 7235–7244. 10.1128/jb.174.22.7235-7244.1992 1429449 PMC207417

[B34] Mueller C. A. Broz P. Müller S. A. Ringler P. Erne-Brand F. Sorg I.. (2005). The V-antigen of Yersinia forms a distinct structure at the tip of injectisome needles. Science 310, 674–676. 10.1126/science.1118476 16254184

[B35] Neyt C. Cornelis G. R. (1999). Insertion of a Yop translocation pore into the macrophage plasma membrane by *Yersinia enterocolitica*: requirement for translocators YopB and YopD, but not LcrG. Mol. Microbiol. 33, 971–981. 10.1046/j.1365-2958.1999.01537.x 10476031

[B36] Nordfelth R. Wolf-Watz H. (2001). YopB of *Yersinia enterocolitica* is essential for YopE translocation. Infect. Immun. 69, 3516–3518. 10.1128/IAI.69.5.3516-3518.2001 11292787 PMC98323

[B37] Olsson J. Edqvist P. J. Bröms J. E. Forsberg Å. Wolf-Watz H. Francis M. S. (2004). The YopD translocator of *Yersinia pseudotuberculosis* is a multifunctional protein comprised of discrete domains. J. Bacteriol. 186, 4110–4123. 10.1128/jb.186.13.4110-4123.2004 15205412 PMC421591

[B38] Persson C. Carballeira N. Wolf-Watz H. Fällman M. (1997). The PTPase YopH inhibits uptake of Yersinia, tyrosine phosphorylation of p130Cas and FAK, and the associated accumulation of these proteins in peripheral focal adhesions. EMBO J. 16, 2307–2318. 9171345 10.1093/emboj/16.9.2307 PMC1169832

[B39] Persson C. Nordfelth R. Andersson K. Forsberg A. Wolf-Watz H. Fallman M. (1999). Localization of the Yersinia PTPase to focal complexes is an important virulence mechanism. Mol. Microbiol. 33, 828–838. 10.1046/j.1365-2958.1999.01529.x 10447891

[B40] Pettersson J. Holmström A. Hill J. Leary S. Frithz-Lindsten E. von Euler-Matell A.. (1999). The V-antigen of Yersinia is surface exposed before target cell contact and involved in virulence protein translocation. Mol. Microbiol. 32, 961–976. 10.1046/j.1365-2958.1999.01408.x 10361299

[B41] Price S. B. Cowan C. Perry R. D. Straley S. C. (1991). The *Yersinia pestis* V antigen is a regulatory protein necessary for Ca2(+)-dependent growth and maximal expression of low-Ca2+ response virulence genes. J. Bacteriol. 173, 2649–2657. 10.1128/jb.173.8.2649-2657.1991 1901573 PMC207833

[B42] Radics J. Königsmaier L. Marlovits T. C. (2014). Structure of a pathogenic type 3 secretion system in action. Nat. Struct. Mol. Biol. 21, 82–87. 10.1038/nsmb.2722 24317488

[B43] Rosqvist R. Bölin I. Wolf-Watz H. (1988). Inhibition of phagocytosis in *Yersinia pseudotuberculosis*: a virulence plasmid-encoded ability involving the Yop2b protein. Infect. Immun. 56, 2139–2143. 3294185 10.1128/iai.56.8.2139-2143.1988 PMC259535

[B44] Rosqvist R. Forsberg A. Wolf-Watz H. (1991). Intracellular targeting of the Yersinia YopE cytotoxin in mammalian cells induces actin microfilament disruption. *Infect*. Immun. 59, 4562–4569. 10.1128/iai.59.12.4562-4569.1991 PMC259078 1937815

[B45] Rosqvist R. Håkansson S. Forsberg A. Wolf-Watz H. (1995). Functional conservation of the secretion and translocation machinery for virulence proteins of yersiniae, salmonellae and shigellae. EMBO J. 14, 4187–4195. 7556059 10.1002/j.1460-2075.1995.tb00092.x PMC394501

[B46] Rosqvist R. Magnusson K. E. Wolf-Watz H. (1994). Target cell contact triggers expression and polarized transfer of Yersinia YopE cytotoxin into mammalian cells. EMBO J. 13, 964–972. 8112310 10.1002/j.1460-2075.1994.tb06341.x PMC394898

[B47] Ryndak M. B. Chung H. London E. Bliska J. B. (2005). Role of predicted transmembrane domains for type III translocation, pore formation, and signaling by the *Yersinia pseudotuberculosis* YopB protein. Infect. Immun. 73, 2433–2443. 10.1128/IAI.73.4.2433-2443.2005 15784589 PMC1087397

[B48] Sasakawa C. Adler B. Tobe T. Okada N. Nagai S. Komatsu K.. (1989). Functional organization and nucleotide sequence of virulence Region-2 on the large virulence plasmid in *Shigella flexneri* 2a. Mol. Microbiol. 3, 1191–1201. 10.1111/j.1365-2958.1989.tb00269.x 2552264

[B49] Sasakawa C. Kamata K. Sakai T. Makino S. Yamada M. Okada N.. (1988). Virulence-associated genetic regions comprising 31 kilobases of the 230-kilobase plasmid in *Shigella flexneri* 2a. J. Bacteriol. 170, 2480–2484. 10.1128/jb.170.6.2480-2484.1988 2836357 PMC211159

[B50] Schiavolin L. Meghraoui A. Cherradi Y. Biskri L. Botteaux A. Allaoui A. (2013). Functional insights into the shigella type III needle tip IpaD in secretion control and cell contact. Mol. Microbiol. 88, 268–282. 10.1111/mmi.12185 23421804

[B51] Simon R. Priefer U. Pühler A. (1983). A broad host range mobilization system for *in vivo* genetic engineering: transposon mutagenesis in gram negative bacteria. Nat. Biotechnol. 1, 784–791. 10.1038/nbt1183-784

[B52] Simonet M. Richard S. Berche P. (1990). Electron microscopic evidence for *in vivo* extracellular localization of *Yersinia pseudotuberculosis* harboring the pYV plasmid. Infect. Immun. 58, 841–845. 2307522 10.1128/iai.58.3.841-845.1990 PMC258544

[B53] Skrzypek E. Straley S. C. (1995). Differential effects of deletions in lcrV on secretion of V antigen, regulation of the low-Ca2+ response, and virulence of *Yersinia pestis*. J. Bacteriol. 177, 2530–2542. 10.1128/jb.177.9.2530-2542.1995 7730287 PMC176914

[B54] Sory M.-P. Cornelis G. R. (1994). Translocation of a hybrid YopE-adenylate cyclase from *Yersinia enterocolitica* into HeLa cells. Mol. Microbiol. 14, 583–594. 10.1111/j.1365-2958.1994.tb02191.x 7885236

[B55] Thorslund S. E. Edgren T. Pettersson J. Nordfelth R. Sellin M. E. Ivanova E.. (2011). The RACK1 signaling scaffold protein selectively interacts with *Yersinia pseudotuberculosis* virulence function. PLoS ONE 6:e16784. 10.1371/journal.pone.0016784 21347310 PMC3037380

[B56] Westermark L. Fahlgren A. Fällman M. (2014). *Yersinia pseudotuberculosis* efficiently escapes polymorphonuclear neutrophils during early infection. Infect. Immun. 82, 1181–1191. 10.1128/IAI.01634-13 24379291 PMC3958004

