# Supplementary material for: The Amino-Terminal Part of the Needle-Tip Translocator LcrV of Yersinia pseudotuberculosis Is Required for Early Targeting of YopH and In vivo Virulence
Source: Front Cell Infect Microbiol. 2016 Dec 5;6:175. doi: 10.3389/fcimb.2016.00175 (PMC5136540; doi:10.3389/fcimb.2016.00175)
Supplement: Supplementary file 1 [file DataSheet1.DOCX]

The Amino-Terminal part of the Needle-Tip Translocator LcrV of *Yersinia pseudotuberculosis* is Required for Early Targeting of YopH and In Vivo Virulence

Sofie Ekestubbe^1,2^, Jeanette E. Bröms^3^, Tomas Edgren^2^, Maria Fällman^1,2^, Matthew S. Francis^2^ and Åke Forsberg^1,2*^

^1^Laboratory for Molecular Infection Medicine Sweden (MIMS), Department of Molecular Biology, Umeå University, Umeå, Sweden

^2^Umeå Centre for Microbial Research (UCMR), Department of Molecular Biology, Umeå University, Umeå, Sweden

^3^Department of Clinical Microbiology, Umeå University, Umeå, Sweden


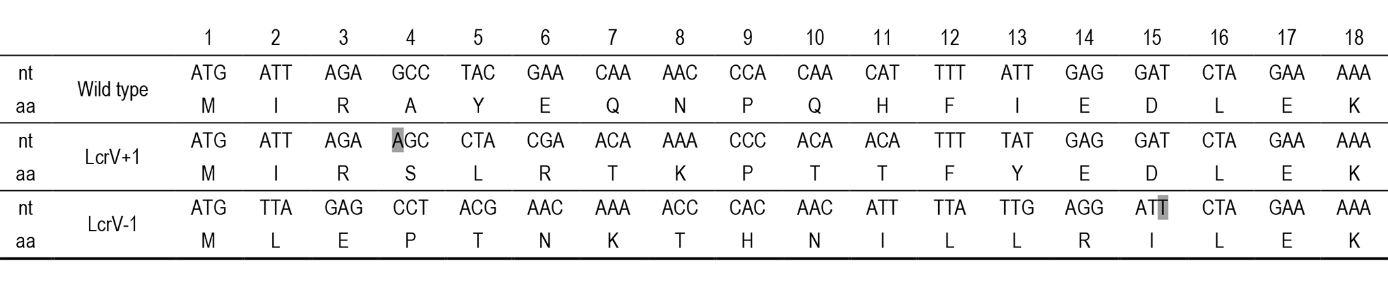


**FIGURE S1. Schematics of the LcrV frameshift mutants. N-terminal n**ucleotide and amino acid sequences are shown for wild type and frameshifted variants of LcrV. The figure is adapted from (Bröms et al., 2007).


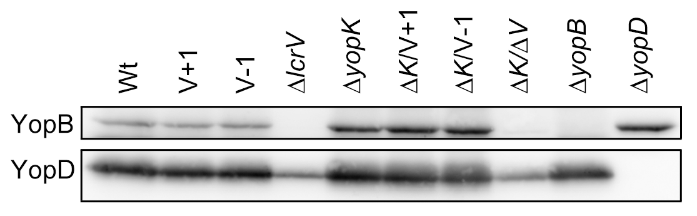


**FIGURE S2. Total expression of YopB and YopD during erythrocyte infection.** Whole cell samples were taken after infection and equal amounts of total protein were subjected to SDS-PAGE and Western blot using an antisera recognizing both YopD and YopB. The experiment was repeated three times and a representative experiment is shown.


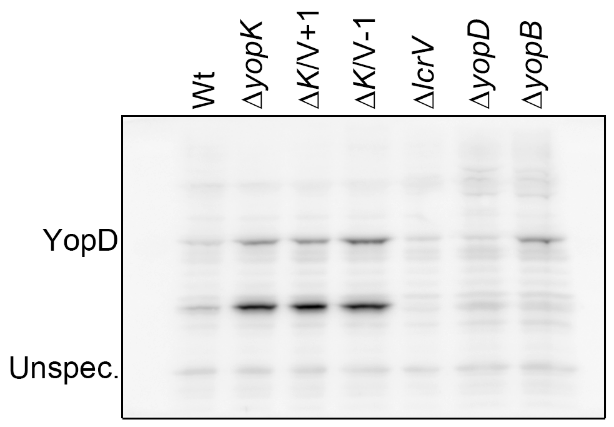


**FIGURE S3. Membrane localization of YopD in HeLa cell membranes.** Original picture of the Western blot of membrane preparation from HeLa cells. The YopD band and the unspecific band used as an internal loading control are indicated.

**TABLE S1.** Oligonucleotides used in this study.

| **Purpose** | **Oligonucleotide pairs** |
| --- | --- |
| YopH_6-468_-Bla TOPO construct | p*yopH*_6_ Forward; 5´-AGCGATCTTCATCGTCAGGTATCT- 3´ and p*yopH*_6-468_-Bla Reverse; 5´-CACCAGCGTTTCTGGGTGGCTATTTAATAATGGTCGC- 3´  p*yopH*_6-468_-Bla Forward; 5´-GCGACCATTATTAAATAGCCACCCAGAAACGCTGGTG- 3´ and pBla Reverse; 5´-TTACCAATGCTTAATCAGTGAGGCACC- 3´ |
| pNQ705-H-Bla | p*yopH*_6_-*Sph*I Forward; 5´-*GCATGC*AGCGATCTTCATCGTCAGGTATCT-3´ and p*bla*-*Sph*I Reverse; 5´-*GCATGC*TTACCAATGCTTAATCAGTGAGGCACC-3´ |

**References**

Bröms, J. E., Francis, M. S., and Forsberg, A. (2007). Diminished LcrV secretion attenuates Yersinia pseudotuberculosis virulence. *J. Bacteriol.* 189, 8417–29. doi:10.1128/JB.00936-07.
